# Abnormal Neuroimaging in a Case of Infant Botulism

**DOI:** 10.3389/fped.2015.00108

**Published:** 2015-12-11

**Authors:** Ryan J. Good, Kevin Messacar, Nicholas V. Stence, Craig A. Press, Todd C. Carpenter

**Affiliations:** ^1^Pediatric Critical Care Medicine, University of Colorado – Denver, Aurora, CO, USA; ^2^Pediatric Infectious Disease, University of Colorado – Denver, Aurora, CO, USA; ^3^Pediatric Neuroradiology, University of Colorado – Denver, Aurora, CO, USA; ^4^Pediatric Neurology, University of Colorado – Denver, Aurora, CO, USA

**Keywords:** infant botulism, neuroimaging, acute flaccid paralysis, toxin, human botulism immune globulin

## Abstract

We present the first case of abnormal neuroimaging in a case of infant botulism. The clinical findings of the patient with constipation, bulbar weakness, and descending, symmetric motor weakness are consistent with the classic findings of infant botulism. Magnetic resonance imaging (MRI), however, revealed restricted diffusion in the brain and enhancement of the cervical nerve roots. Traditionally, normal neuroimaging was used to help differentiate infant botulism from other causes of weakness in infants. Abnormal neuroimaging is seen in other causes of weakness in an infant including metabolic disorders and hypoxic–ischemic injury, but these diagnoses did not fit the clinical findings in this case. The explanation for the MRI abnormalities in the brain and cervical nerve roots is unclear as botulinum toxin acts at presynaptic nerve terminals and does not cross the blood–brain barrier. Possible explanations for the findings include inflammation from the botulinum toxin at the synapse, alterations in sensory signaling and retrograde transport of the botulinum toxin. The patient was treated with human botulism immune globulin and had rapid recovery in weakness. A stool sample from the patient was positive for Type A *Clostridium botulinum* toxin eventually confirming the diagnosis of infant botulism. The findings in this case support use of human botulism immune globulin when the clinical findings are consistent with infant botulism despite the presence of MRI abnormalities in the brain and cervical nerve roots.

## Introduction

A 5-month-old male infant presented with progressive weakness. Two weeks prior to presentation, the infant developed constipation that improved with stool softeners. He subsequently developed difficulty feeding, ptosis, and weakness in his trunk and extremities. On presentation, the infant was afebrile with age-appropriate vital signs. Cranial nerve exam was notable for bilateral ptosis and a weak grimace. Motor exam was notable for diffuse appendicular and axial hypotonia with significant head lag and a positive scarf sign. The infant was born at term with no significant past medical history. His diet consisted of primarily breast milk with no exposure to honey or well water. Social history was notable for residence in a farming community in Western Nebraska. Results of his newborn screen were unavailable, but his parents were not aware of any abnormalities.

Initial evaluation including complete blood count, complete metabolic panel, lactate, and urinalysis was unremarkable. Metabolic screening labs, including serum amino acids, acylcarnitine profile, and urine organic acids, were normal. Cerebrospinal fluid (CSF) had 0 white blood cells, 0 red blood cells, protein of 21 mg/dL, and glucose of 65 mg/dL. A nasopharyngeal sample tested positive by multiplex viral polymerase chain reaction (PCR) for rhino/enterovirus. Enterovirus PCR testing of CSF, throat, and rectal swabs was negative.

**Figure 1:**
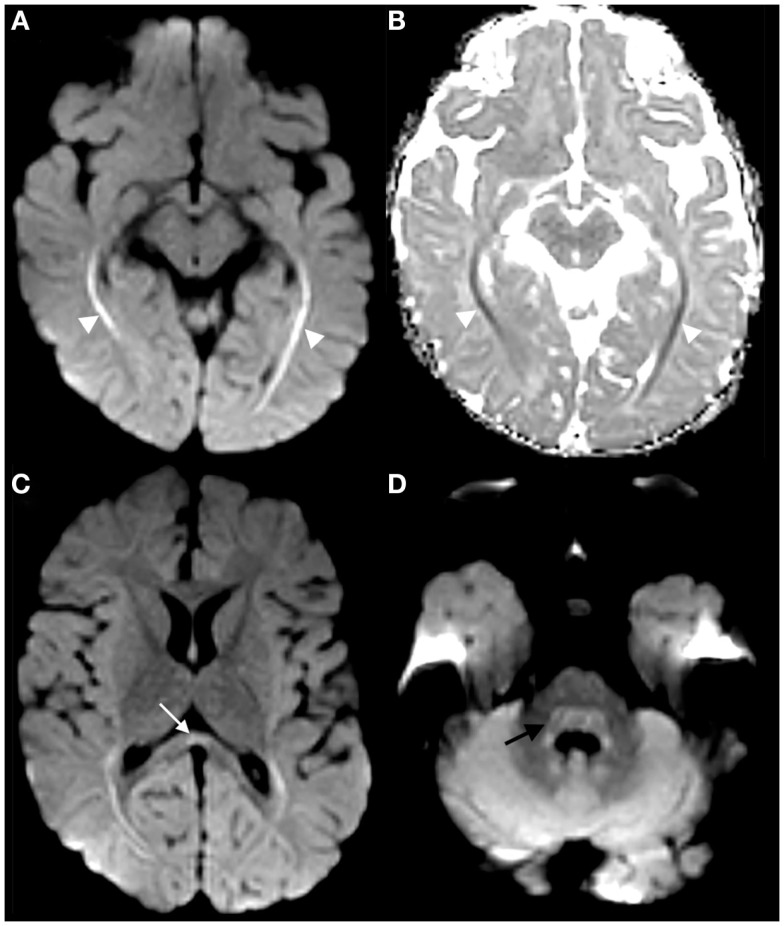
**Trace diffusion-weighted image of the brain demonstrates hyperintensity along the optic radiations [arrowheads in (A)], with corresponding hypointensity on ADC map [arrowheads in (B)], compatible with restricted diffusion**. Restricted diffusion was also faintly present in the corpus callosum splenium [arrow in **(C)**] and dorsal pons [curved arrow in **(D)**].

**Figure 2:**
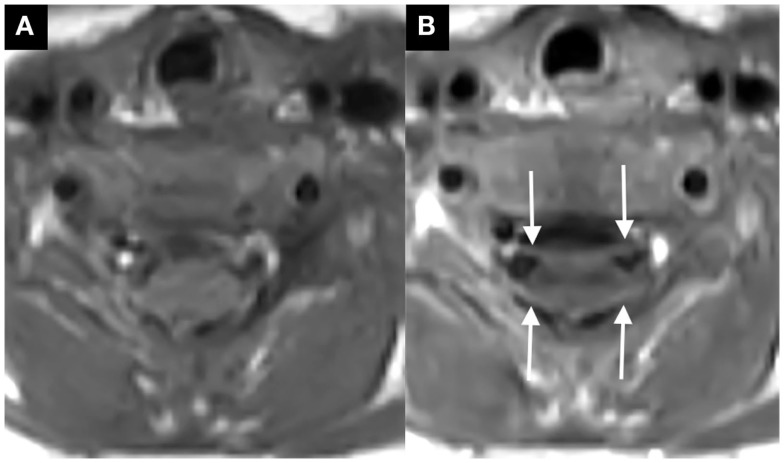
**Axial post-contrast T1-weighted image through the cervical spine, ventral, and dorsal cervical nerve roots abnormally enhance [thin arrows in (B), compare to non-contrast image at same level in (A)]**.

The infant was admitted to the Pediatric Intensive Care Unit for respiratory monitoring and further evaluation. Magnetic resonance imaging (MRI) of the brain and spine was performed on a 3-T Philips Ingenia magnet. Brain diffusion-weighted imaging demonstrated restricted diffusion of the corpus callosum, splenium, bilateral optic radiations, and dorsal pons (Figures 1A–D). There was no abnormal brain parenchyma or cranial nerve enhancement. Magnetic resonance spectroscopy over the optic radiations was normal (not shown). MRI of the spine revealed symmetric cervical nerve root enhancement without spinal cord signal abnormality (Figures 2A,B). Electrophysiological studies were not performed.

## Background

Infant botulism is a toxin-mediated infectious disorder that causes constipation, bulbar weakness, and descending, symmetric motor weakness (1). Over 3,000 cases of infant botulism have been reported since 1976. Most of these cases have been reported in the US with 80–100 cases annually (2). The diagnosis of infant botulism must be made clinically for timely administration of botulism immune globulin (BabyBIG) and later confirmed by a *Clostridium botulinum* mouse bioassay. Sensitivity of clinical diagnosis of infant botulism has not been studied, but Arnon et al. reported that 122 of 129 patients with clinical suspicion for infant botulism treated with BabyBIG later had laboratory confirmation of the disease (3). Treatment with BabyBIG decreases length and cost of hospital stay as well as severity of illness but is most effective when given early in the disease course. The findings in this case including constipation, bulbar palsy, and descending, symmetric motor weakness are consistent with symptoms in other cases of infant botulism.

## Discussion

Given the history and exam consistent with infant botulism along with CSF reassuring against infectious or autoimmune etiologies for the infant’s weakness, the infant was treated empirically with BabyBIG on hospital day 2 despite the abnormal neuroimaging. After treatment with BabyBIG, the infant had rapid improvement in weakness and was discharged on hospital day 5. A stool sample was obtained via enema prior to discharge and sent for *C. botulinum* mouse bioassay at the Centers for Disease Control and Prevention that later confirmed the presence of Type A *C. botulinum* toxin. On phone follow-up 6 months after admission, the parents reported that the patient continued to have constipation but no other residual neurological problems. The parents declined follow-up imaging despite being made aware of the abnormalities present on the initial study.

This is the first reported case of infant botulism associated with abnormal MRI findings in the brain and cervical nerve roots. The findings may be incidental but support the use of BabyBIG in infants with suspected infant botulism despite neuroimaging abnormalities. Reviews of infant botulism do not discuss neuroimaging findings as part of the diagnosis and several recent case reports of infant botulism reported normal brain MRI findings (1, 4–7). Attenuated cortical thickening over the frontal and temporal lobes has been described in one case report of infant botulism, but this imaging was done by head computed tomography (CT) during the recovery phase of the illness in a patient with persistent neurological abnormalities (8). Normal neuroimaging has traditionally been used to help differentiate infant botulism from alternative causes of acute flaccid paralysis in the infant, including demyelinating diseases, metabolic disorders, ischemia, spinal cord compression, and neoplastic process.

Enterovirus infection causing flaccid paralysis was considered in this case given the nasal swab PCR positive for rhino/enterovirus, but the history of constipation and descending weakness, the diffusion-weighted changes on brain MRI, the lack of spinal cord signal abnormality, and the rapid improvement after BabyBIG administration was not consistent with the recently described association of enterovirus D68 and acute flaccid myelitis (9, 10). Other causes of weakness in an infant could cause neuroimaging abnormalities similar to those seen in this case but were similarly inconsistent with the clinical course. Metabolic disorders, mitochondrial disease, and leukodystrophies can cause central and peripheral nerve injury and demonstrate symmetric patterns of central restricted diffusion. These diseases, however, would not respond to BabyBIG and would be expected to causes ongoing clinical symptoms. Additionally, the absent lactate peak on spectroscopy over the area of restricted diffusion would be atypical in the acute imaging of mitochondrial disease. Hypoxic–ischemic injuries can cause white matter restricted diffusion, but this case lacked a history suggestive of hypoxic injury and the pattern of imaging findings, specifically the cervical nerve root enhancement, would be atypical.

It is possible that the neuroimaging abnormalities found in this case are not unique for cases of infant botulism but have not been previously detected as imaging is infrequently acquired and ongoing improvements in MRI techniques and technology may have made the findings more obvious. The abnormal enhancement of the cervical nerve roots correlated with the bulbar weakness seen in the patient. The potential mechanism of nerve root enhancement is puzzling since botulinum toxin acts at presynaptic nerve terminals. One explanation is that the finding represents inflammation of the nerve root caused by either the neurotoxin at the synapse or by retrograde signaling. It is more difficult to explain the brain diffusion-weighted findings, as botulinum toxin does not cross the blood–brain barrier. Botulinum toxin specificity is derived from its binding to specific gangliosides and other receptors on the presynaptic nerve terminal (11). Once endocytosis occurs, it is possible that retrograde transport could lead to direct effects on the CNS, and studies have suggested that botulinum toxin may inhibit neurotransmitters other than acetylcholine. Other studies have shown that altered sensory input produced by botulinum toxin can cause indirect changes in the central nervous system (12). It is unclear if the brain diffusion-weighted findings in this case could be a direct effect of the toxin through retrograde transport, an indirect effect on peripheral and sensory nerves, or incidental.

## Concluding Remarks

We report the first known case of abnormal neuroimaging findings in a case of infant botulism. Although unusual for this disease, abnormal MRI findings should not absolutely exclude this diagnosis in the setting of other characteristic clinical findings. The findings in this case support treatment of suspected infant botulism with BabyBIG despite the unusual neuroimaging findings of restricted diffusion in the brain and cervical nerve root enhancement.

## Consent

The author obtained parental consent for publication of this case report prior to submission.

## Author Contributions

RG drafted the initial manuscript and approved the final manuscript as submitted. NS provided the figures for the manuscript, reviewed and revised the manuscript, and approved the final manuscript as submitted. KM, CP, and TC reviewed and revised the manuscript and approved the final manuscript as submitted. All authors approved the final manuscript as submitted and agree to be accountable for all aspects of the work.

## Conflict of Interest Statement

The authors declare that the research was conducted in the absence of any commercial or financial relationships that could be construed as a potential conflict of interest.
